# Recall T cell responses to bluetongue virus produce a narrowing of the T cell repertoire

**DOI:** 10.1186/s13567-017-0444-3

**Published:** 2017-06-29

**Authors:** José-Manuel Rojas, Teresa Rodríguez-Calvo, Noemí Sevilla

**Affiliations:** 1 Centro de Investigación en Sanidad Animal (CISA-INIA), Instituto Nacional de Investigación Agraria y Alimentaria, Ctra Algete a El Casar km 8, Valdeolmos, 28130 Madrid, Spain; 2 Institute of Diabetes Research, Helmholtz Zentrum München, Deutsches Forschungszentrum für Gesundheit und Umwelt (GmbH), Neuherberg, Germany

## Abstract

In most viral infections, recall T cell responses are critical for protection. The magnitude of these secondary responses can also affect the CD8 and CD4 epitope repertoire diversity. Bluetongue virus (BTV) infection in sheep elicits a T cell response that contributes to viremia control and could be relevant for cross-protection between BTV serotypes. Here, we characterized CD4^+^ and CD8^+^ T cell responses during primary and recall responses. During primary immune responses, both CD4^+^ and CD8^+^ T cell populations expanded by 14 days post-infection (dpi). CD4^+^ T cell populations showed a lower peak of expansion and prolonged contraction phase compared to CD8^+^ T cell populations. Recall responses to BTV challenge led to BTV-specific expansion and activation of CD8^+^ but not of CD4^+^ T cells. The evolution of the BTV-specific TCR repertoire was also characterized in response to VP7 peptide stimulation. Striking differences in repertoire development were noted over the time-course of infection. During primary responses, a broader repertoire was induced for MHC-I and MHC-II epitopes. However, during memory responses, a narrowed repertoire was activated towards a dominant motif in VP7 comprising amino acids 139–291. Monocytes were also examined, and expanded during acute infection resolution. In addition, pro-inflammatory cytokine levels increased after BTV inoculation and persisted throughout the experiment, indicative of a prolonged inflammatory state during BTV infections. These findings could have implications for vaccine design as the narrowing memory T cell repertoire induced after BTV re-infection could lead to the development of protective immunodominant TCR repertoires that differs between individual sheep.

## Introduction

The role of memory T cells is to help protect the host during secondary antigen encounters. These secondary responses can reinforce the quantity and quality of the immune response against the challenging pathogen [1]. This is reflected by the increased frequency of antigen-specific T cells able to mount accelerated responses, with shorter antigenic stimulation leading to efficient and quicker antigen clearance. However, T cell exhaustion can also occur in the presence of saturated levels of antigen or even after repetitive natural or vaccination exposures. In particular, an understanding of T cell recruitment/expansion process during the recall response may have significant implications for effective control strategies. Different mechanisms of memory recruitment have been postulated. A memory population in which highly effective clones predominate may occur as a stochastic expansion, more likely maintaining the T cell diversity, which was shown to be beneficial for virus control [2]. Alternatively, by deterministic selection, high quality clones in the memory population may be expanded by antigen-driven mechanisms [3], narrowing the T cell repertoire [4].

Bluetongue virus (BTV) is the prototype member of the genus *Orbivirus* within the *Reoviridae* family, transmitted by Culicoides midges [5]. BTV infects ruminants, causing an acute disease with high morbidity and mortality [6]. The BTV genome is composed of 10 segments of double-stranded RNA encoding 4 non-structural and 7 structural proteins that is enclosed by a complex capsid structure [7, 8]. There are at least 27 serotypes circulating based on the specific neutralizing antibodies raised against VP2 [9–11]. The immune response against BTV is characterized by the induction of humoral responses, neutralizing antibodies, and cellular immunity that contributes significantly to protection in vaccinated animals [12–15]. Virus-specific CD8^+^ cytotoxic T lymphocytes (CTL) are key components of the immune response, inducing cross-protection among different serotypes [16, 17]. In nature, high frequency of repeated BTV infections due to successive bites by biting midges may occur, meaning successive challenges with other BTV serotypes (heterologous virus) or with the same serotype (homologous virus).

Immune responses to viral infections are not mounted in immunological isolation, as the immune response to one virus may condition the host to elicit an altered immune response against a homologous or heterologous virus. Using successive challenge with BTV-8, we investigated the expansion of VP7-specific CD8^+^ and CD4^+^ T cells [18]. Furthermore, we studied the inflammatory response during recall responses. Here, we show that recall responses with BTV led to BTV-specific expansion and activation of CD8^+^ but not of CD4^+^ T cells. Interestingly, during primary responses, a broader repertoire of T cell epitopes was induced. However, during memory responses, a narrowed repertoire was activated towards a dominant epitope in VP7.

## Materials and methods

### Virus

A BTV-8 isolate (Belgium/06) from an infected calf in the 2006 Belgium outbreak was used in this study [19]. BTV-8 was expanded in baby hamster kidney (BHK) cells (ATCC CCL-10) and titered in semi-solid agar medium in Vero cells (ATCC CCL-81) as described [20]. BTV-8 inactivation with binary ethylenimine (BEI) was performed as described [21].

### Animals and experimental design

Three-month old BTV naive female Mallorquina sheep were kept in a disease-secure isolation facility (BSL3) at the Centro de Investigación en Salud Animal (CISA), in strict accordance with the recommendations in the guidelines of the Code for Methods and Welfare Considerations in Behavioural Research with Animals (Directive 86/609EC; RD1201/2005) and all efforts were made to minimize suffering. Experiments were approved by the Committee on the Ethics of Animal Experiments (CEEA) (Permit Number: 10/142792.9/12) of the Spanish Instituto Nacional de Investigación y Tecnología Agraría y Alimentaria (INIA) and the ‘‘Comisión de ética estatal de bienestar animal’’ (Permit Numbers: CBS2012/06 and PROEX 228/14). An acclimatization period of 2 weeks was observed, during which the animals were monitored daily for general health status prior to the experiment.

Animals (*n* = 8) were inoculated subcutaneously with 1 × 10^5^ pfu BTV-8 three times at 28 day intervals. Two naive controls were inoculated with PBS at the same time points as the control group.

### Peripheral blood mononuclear cell isolation

Venous blood from BTV-8 infected sheep was collected on days 0, 7, 14, 28, 35, 56, 63 and 70, and PBMC were isolated by standard centrifugation methods on Ficoll [18]. Flow cytometry studies were performed on freshly isolated PBMC. Remaining PBMC were frozen and stored in liquid nitrogen until use.

### Peptides and antibodies

Peptides from BTV-8 VP7 protein (ACJ06230) were selected as described in [18] and synthesized by Altabiosciences (Birmingham, UK) (Table 1 corresponds to oligonucleotide sequences). All peptides were resuspended in DMSO and stored at −80 °C. The following directly conjugated antibodies were used in this study: anti-sheep CD4 (44.38); anti-sheep CD8 (38.65); anti-human (cross-reactive with sheep) CD14 (TÜK4) (all from Biorad); anti-BTV-VP7 (CF-J-BTV-MAB-10ML).

**Table 1:** Primer sequences for real-time RT-PCR

Gene	Forward (5′–3′)	Reverse (5′–3′)
IL-6	CCTCCAGGAACCCAGCTATG	GGAGACAGCGAGTGGACTGAA
IL-1β	CGAACATGTCTTCCGTGATG	TCTCTGTCCTGGGAGTTTGCAT
IL-12	CGTGATGGAAGCTGTGCAC	CTTTCCTGGACCTGAACAC
CXCL10	GCTCATCACCCTGAGCTGTT	AGCTGTCAGTAGCAAGGCTA

### Flow cytometry

Surface flow cytometry stainings were performed using staining buffer (PBS + 2% FBS + 0.05% sodium azide). PBMC were washed twice, stained with antibodies for 20 min on ice, and finally washed twice in staining buffer. For BTV-VP7 intracellular staining, PBMC were fixed in 4% paraformaldehyde for 10 min, permeabilized and washed 3 times in staining buffer containing 0.2% saponin and incubated for 30 min on ice with antibody diluted in staining buffer supplemented with 0.2% saponin. For all stainings appropriate isotype and fluorescence minus one controls were included. Acquisitions were performed on a BD FACScalibur flow cytometer and analysis using FlowJo software (Tree Star Inc, USA).

### RNA isolation, reverse transcription and quantitative real-time PCR to determine viral load

RNA from total blood was obtained using Trizol Reagent Solution (Thermo Fisher Scientific) and following the manufacturer’s protocol. Viral RNA loads were determined by amplification of segment 5 as described [12, 22] using the QIAGEN OneStep RT-PCR kit (QIAGEN) or the Ambion AgPath-ID One-Step RT-PCR (Thermo Fisher Scientific).

### Expression of immune genes by real time PCR

Total RNA was extracted from PBMC isolated at different times post-infection using Trizol Reagent Solution (Thermo Fisher Scientific). Isolated RNA was treated with DNase I (BioLabs New England) according to the manufacturer`s protocol. 1 μg of RNA was used to obtain cDNA using the SuperScript™ II reverse Transcriptase (Thermo Fisher Scientific) and oligo (dT)_12–18_ (0.5 μg/mL). To evaluate the levels of transcription of IL-12, IL-6, CXCL10 and IL-1β, real time was performed in a LightCycler 480 System instrument (Roche) using SYBR Green PCR core Reagents (Applied Biosystems) and specific primers (Table 1). Each sample was measured under the following conditions: 10 min at 95 °C followed by 45 amplification cycles (15 s at 95 °C and 1 min at 60 °C). The expression of individual genes was normalized to relative expression of ovine GPDH gene and the expression levels were calculated using 2^−ΔCt^ method, where ΔCt is determined by subtracting the GPDH value from the target Ct. A melting curve for each PCR fluorescence reading, every degree between 60 and 95 °C, was determined to ensure that only a single product had been amplified.

### ELISA for sheep IFN-γ

PBMC (2 × 10^5^ per well) were stimulated for 24 h in U-bottom 96 well plates with BEI-BTV-8 (equivalent to 1 × 10^5^ pfu prior to inactivation), concanavalin-A (2.5 µg/mL) as the positive control, VP7 peptide (10 µg/mL), or an equivalent volume of DMSO as the negative control. IFN-γ production in culture supernatants was then tested using a commercially available IFN-γ ELISA kit (Mabtech, Sweden). The ELISA detection limit was 20 pg/mL. Data were normalized to 1 × 10^6^ PBMC.

### Statistical analysis

Statistical analysis was performed using Prism 5.0 software (Graphpad Software Inc, USA). Levels of significance were *p < 0.05; **p < 0.01; ***p < 0.001.

## Results

### Clinical responses

**Figure 1:**
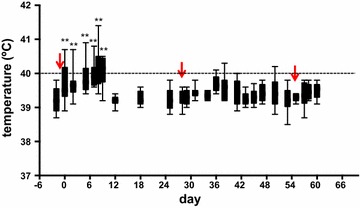
**Temperature measurements after primary and recall BTV-8 infections in sheep.** Sheep rectal temperatures were measured prior to BTV-8 sc inoculation and after primary and recall infections (indicated by red arrows). ** *p* < 0.01; one-way ANOVA test with Bonferroni’s post-test (timepoints vs day −2).

In order to evaluate and compare the expansion of the immune response after primary and secondary BTV infections, one in vivo experiment with 10 animals was done, in which 8 sheep were inoculated sc with BTV-8 three times at 28-day intervals (see “Materials and methods”). All animals inoculated with BTV-8 developed clinical signs and fever (> 40 °C during four successive days) at days 5–8 post-infection (Figure 1). The first sign of the disease was a slight to moderate increase in respiratory rate, accompanied by inflammation of the oral mucosa. After day 9, temperature declined to normal levels and disease signs were detectable up to day 15, achieving full recovery afterwards. BTV-8 inoculation at day 28 and 56 did not increase rectal temperature nor produce clinical signs, suggesting that an efficient BTV-specific immune response had been elicited in BTV-infected sheep. Control PBS-inoculated sheep did not show any clinical signs or fever during the experiment.

### Expansion of PBMC after challenge with BTV in primary and secondary responses

**Figure 2:**
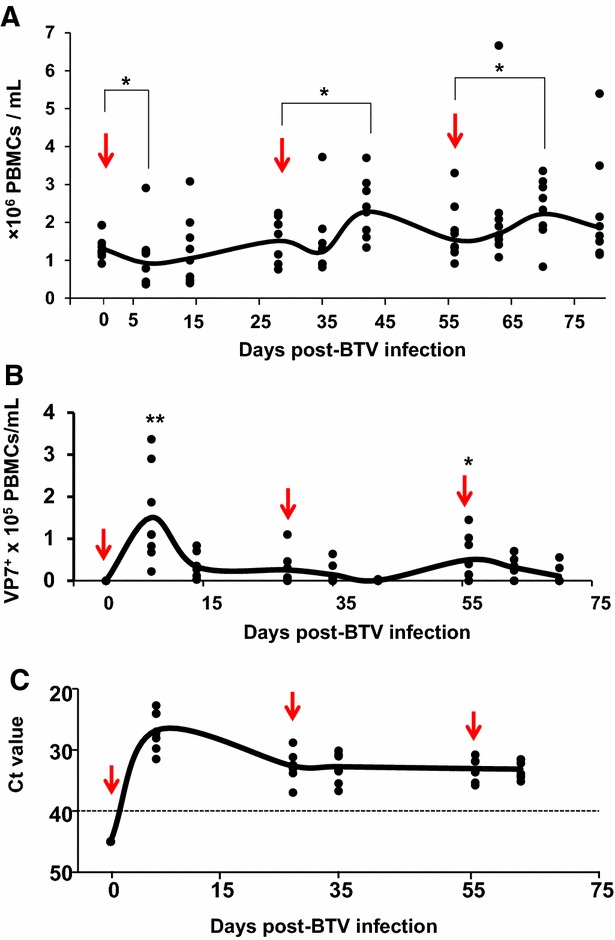
**Expansion of PBMC and viral load during acute and recall BTV infection.** Sheep were inoculated with BTV-8 three times at 28 day intervals (see “Materials and methods” section) and bled at different times post-inoculation. **A** Average count of PBMC relative to pre-BTV infection at different days post-inoculation. The arrow indicates the inoculation day. * *p* < 0.05; Mann-Whitney test (timepoint vs inoculation time). **B** Number of PBMC/mL of blood that were positive for VP7 by flow cytometry staining at different days post-BTV infection. Arrows indicate inoculation days. * *p* < 0.05, ** *p* < 0.01; Mann–Whitney test (timepoint vs day 0). **C** Whole blood was collected at different days post-BTV infection. Total RNA was extracted and RT-qPCR for BTV segment 5 was performed as indicated in the “Materials and methods”. The results are expressed as Ct. The cut-off is indicated with a dotted lined (Ct = 40 according to [22]).

To evaluate the immune response after primary and secondary BTV infections, and according to the scheme indicated above, total PBMC were isolated from peripheral blood and quantified at different times post-inoculation. During primary response, a significant decrease in the number of PBMC was detected at day 7 post-infection (D7pi) returning to basal levels by D14pi (Figure 2A). Twenty-eight days later, sheep were inoculated with the same amount of BTV-8 sc (secondary BTV infection) and the number of PBMC was determined. In contrast to the primary infection, PBMC levels did not decrease 7 days after the second infection (D35pi) and significantly increased 14 days after this second infection (D42pi), followed by a contraction period. Similar results were obtained after the third challenge. Because BTV may infect lymphocytes causing cell death [23], we next asked whether BTV infection of PBMC would result in PBMC depletion after primary infection. PBMC were labeled with a specific monoclonal antibody against the VP7 protein and directly coupled to a fluorochrome probe. A significant amount of PBMC were positive for VP7 protein by flow cytometry analysis (average of 1.51 × 10^5^ ± 0.2 PBMCs/mL from 8 sheep infected with BTV-8) by D7pi (Figure 2B), suggesting that the D7pi decrease in PBMC numbers might be due to BTV-induced cell death. The number of VP7 positive cells did not increase after secondary infection but it was maintained until at least D45pi. After the third infection, a significant increase of VP7 positive PBMC was observed, suggesting that a brief round of BTV infection occurred shortly after inoculation (D3pi). This contrasts with primary infection where the peak of VP7 positive cells was obtained at D7pi. To assess virus replication, blood samples for each sheep were examined for viral genome by RT-qPCR. A peak in viremia was found at D7pi (average Ct value of 26.82 ± 3) followed by a slow reduction in circulating virus. Importantly, the infection was not completely cleared by the end of the experiment (average Ct value 33.1 ± 1.4 at D63pi) (Figure 2C). New viremia peaks were nonetheless never detected after secondary or tertiary challenge. Taken together these data suggest that during secondary BTV infections PBMC expanded but BTV infection was not completely controlled.

### Re-exposure to virus controls the magnitude of the CD4 and CD8 T cell responses

**Figure 3:**
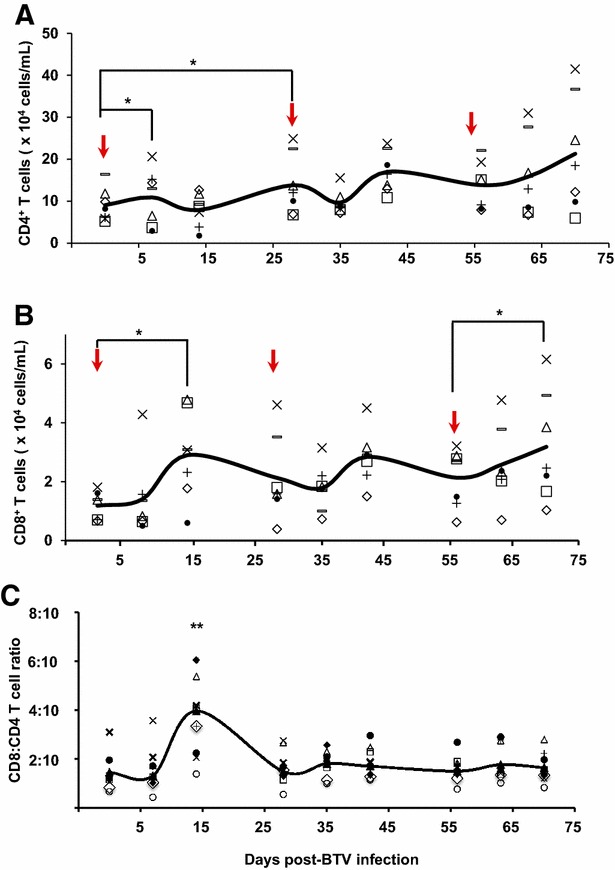
**CD4** ^**+**^ **and CD8** ^**+**^ **T cell expansion during acute and recall BTV infection.** PBMC were obtained at the indicated time points after primary, secondary or tertiary BTV infection (indicated by an arrow) and staining for CD4 and CD8-T cell was done by flow cytometry (see “Materials and methods”). **A** Number of CD4^+^-T cells over time in PBMC of individual sheep. * *p* < 0.05; Mann-Whitney test (timepoints vs inoculation time). **B** Number of CD8^+^-T cells in PBMC over time of individual sheep. * *p* < 0.05; Mann-Whitney test (time points vs inoculation time). **C** Kinetics of specific CD8:CD4 T-cell ratio. The number of CD8^+^ T cells was divided by the number of CD4^+^ T-cells during the time course of infection. ** *p* < 0.01; Mann-Whitney test (day 14 vs all timepoints).

Given that total PBMC numbers expanded 14 days after acute (D14pi vs D7pi) and secondary infections with BTV (D42pi vs D28pi and D56pi vs D70pi), we examined whether an increase in CD4^+^ or CD8^+^ T cell populations could explain this expansion. CD4^+^ T cell numbers started to increase significantly at D7pi, maintaining this trend up to D28pi (Figure 3A). Surprisingly, the population of CD4^+^ T cells slightly declined 7 days after the second infection (D35pi) but increased 15 days later (D42pi). After the third BTV challenge, the CD4^+^ T cell population did not show any significant changes in numbers although at D15 post-third infection (D70pi) CD4^+^ T cell numbers slightly increased. In general, CD8^+^ T cell responses peak at about 1 week post-infection in most viral infections, and soon thereafter, virus-specific T cells eliminate the virus [24, 25]. Interestingly, CD8^+^ T cell responses peaked at D14 post-BTV challenge both in acute (D14pi) and secondary responses (D42pi and D70pi) (Figure 3B). CD8^+^ T cells thus proliferated in response to the virus, independently of primary or secondary infections. The decrease in CD8^+^ T cell numbers following D15pi expansion led to a subsequent significant decrease in the CD8:CD4 T cell ratio (Figure 3C). Although repeated infections slightly increased CD8^+^ T cell numbers (D42pi and D70pi), CD4:CD8 T cell ratios were unchanged as CD4^+^ T cell numbers slightly increased concomitantly. This unchanged CD8:CD4 T cell ratio indicated that CD8^+^ T cells did not significantly proliferate after antigen re-exposure. Thus, our findings indicate that levels of central CD8^+^ memory T cells did not stabilize after primary infection, suggesting that in the absence of further infections the memory level of CD8^+^ T cells would continually decline.

### Characterization of T cell responses after primary and secondary infections

**Figure 4:**
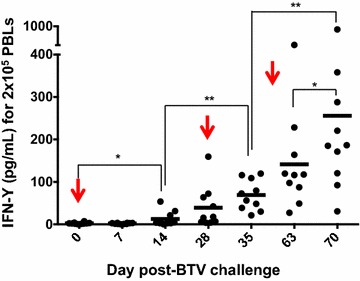
**Kinetics of IFN-γ production by activated T cells.** PBMC from BTV-infected sheep were stimulated with BTV-BEI inactivated (see “Materials and methods” section) and the amount of IFN-γ produced was evaluated by ELISA. The arrows indicate days of BTV inoculation. * *p* < 0.05, ** *p* < 0.01; Mann-Whitney test.

We next sought to analyze the BTV-specific CD4^+^ and CD8^+^ T cell responses. The global T cell responses were first evaluated to determine differences in the overall magnitude of the responses to primary and secondary BTV infections. To this end, PBMC from BTV-infected sheep were isolated at different times post-infection, stimulated in vitro with inactivated-BTV-8, and IFN-γ production was evaluated by ELISA. A significant production of IFN-γ was found at D15pi after primary infection (D0) (Figure 4). Recall infections also induced significant IFN-γ responses 7 and 15 days after the second or third challenge (D35pi, D63pi and D70pi). In addition, the amplitude of these T cell responses increased over time, suggesting that there was a prolonged activation of T cell responses during the observed period, probably due to inefficient virus clearance that might continuously activate CD8^+^ T cells responses.

**Figure 5:**
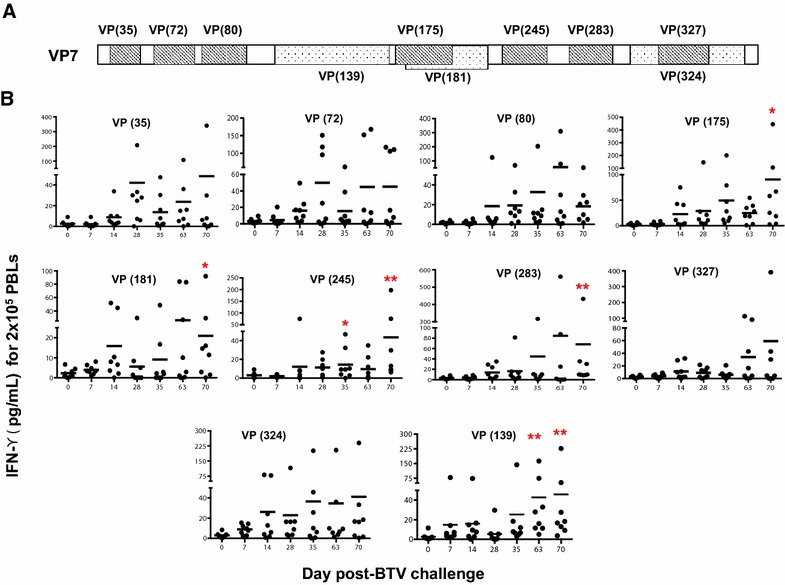
**Differences in the TCR repertoire of CD4 and CD8 T cells specific to BTV VP7. A** VP7 amino acid sequence of BTV VP7 protein showing the previously described T cell epitopes [18]. Striped boxes indicate MHC-I epitopes and dotted boxes indicate MHC-II epitopes. The name of each epitope is indicated with VP and the position of the first amino acid for each epitope in VP7. **B** PBMC isolated from BTV-infected sheep at different days post-infection were stimulated with individual peptides (indicated on each graph) and the amount of IFN-γ produced by T cells was evaluated by ELISA. * *p* < 0.05, ** *p* < 0.01; Mann-Whitney test (timepoints vs day 0).

T cell recognition of infected cells is accomplished by the interaction of clonally distributed TCR on effector cells with the complex of viral peptide/MHC class I-II on infected cells [26, 27]. The specificity of T cells for a particular MHC/peptide combination determines, at least in part, the TCR repertoires within an antigen-specific population. In order to determine the spectrum of the TCR repertoire against VP7 during primary and recall responses, PBMC from BTV-infected sheep were stimulated in vitro with peptides that comprise the main T cell epitopes in VP7 protein (Figure 5A) [18], and the production of IFN-γ was measured by ELISA. During acute infection, most T cells synthesized IFN-γ in response to most of the epitopes (D7pi and D14pi) (Figure 5B), showing a broad distribution of TCR diversity. This was also observed after secondary response, in which T cell responses to all the epitopes were detected. However, by D70pi, after the third BTV challenge, a T cell response bias towards 3 MHC-I epitopes [VP(175), VP(245) and VP(283)] and 2 MHC-II epitope [VP(139) and VP(181)] became apparent. All these epitopes were grouped in a cluster of 100 amino acids of VP7, indicating a restriction in the TCR diversity that responds to BTV after repeated infections and thereby a narrowing of the TCR repertoire during memory responses.

### Persistent inflammation during acute and secondary responses

**Figure 6:**
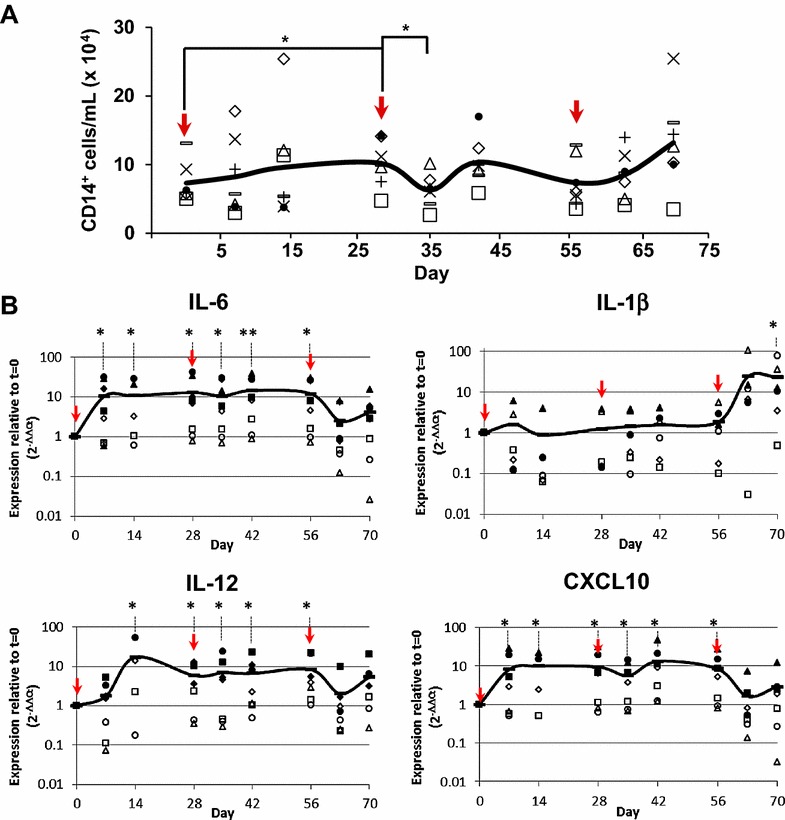
**Inflammatory response during BTV infection. A** CD14^+^ cells were quantified by flow cytometry from total PBMC obtained from BTV-infected sheep. **B** RNA was extracted from PBMC from BTV-infected sheep at different times post-infection. Reverse transcription to cDNA and IL-6, IL-1β, IL-12 and CXCL10 expression was assessed by RT-qPCR. The line represents the average gene expression in 4–8 animals tested at each time point. The arrows indicate time of infection with BTV-8. Data were normalized to β-actin expression and to pre-infection values (t = 0) (2^−∆∆Ct^ method). Student t test (time point vs t = 0); * *p* ≤ 0.05, ** *p* ≤ 0.01.

Upon virus infection, monocytes play a role in initiating the adaptive immune response, and affect Th1/Th2 polarization by producing proinflammatory and immune-modulatory cytokines [28]. Therefore, we studied the magnitude of the monocyte response during primary and secondary BTV responses by flow cytometry in PBMC from sheep infected with BTV. Analysis of CD14-expressing cells (Figure 6A) revealed a steady increase in CD14^+^ monocytes during infection. This increase was transient and started to decline by D28pi, right after the second challenge with BTV. By day 15 after the second BTV challenge (D42pi), CD14^+^ cell numbers increased followed by a progressive decline by D56pi. After the third BTV challenge (D56pi), the population of CD14-expressing cells increased until the experiment ended. To study the cytokine/chemokine response during BTV infection, we determined the amount of mRNA expressed by PBMC of BTV-infected sheep at different time points by RT-qPCR. IL-6 and IL-1β were chosen as representative pro-inflammatory cytokines [29, 30]. IL-6 transcription was significantly up-regulated during primary infection, starting at D7pi (Figure 6B) and during the course of the second BTV challenge. However, after D56pi, IL-6 mRNA transcripts declined to levels similar to prior BTV infection. In contrast, IL-1β mRNA levels showed an opposite transcription pattern to IL-6. IL-1β mRNA was not up-regulated until the third challenge, by D56pi (Figure 6B). In addition to these pro-inflammatory cytokines, transcript levels of IL-12, a classical Th1 cytokine [31] and CXCL10, a chemokine that mediates leukocyte trafficking and activates Th1 responses (reviewed in [32]) were evaluated. Both cytokines showed a significant up-regulation of transcription during infection until D56pi (Figure 6B). Thus, IL-6, IL-12 and CXCL10 were significantly up-regulated after primary and secondary responses while IL-1β was up-regulated only after D56pi, suggesting that an active inflammatory response was induced during primary and secondary BTV infection that did not involve IL-1β.

## Discussion

In this report we analyzed the T cell immune response elicited in sheep after repetitive infections with BTV-8, mimicking several peaks of vector activity during the course of the year [33]. Our results show that although an expansion of PBMC occurs after serial BTV inoculations, the infection is not completely controlled, resulting in detectable BTV RNA by RT-qPCR. Similarly, in BTV-8-immunized calves BTV1/15 replication still occurred after heterologous challenge [34]. In our study, the characterization of the expansion of CD4^+^ and CD8^+^ T cell responses during primary and secondary responses demonstrated a burst in size of the CD8^+^ but not the CD4^+^ population 15 days after each challenge. Moreover, IFN-γ production was also detected 15 days after primary and secondary challenge, although the TCR repertoires present within primary responses and secondary recall responses were different. These findings indicate that during BTV reinfections there is a bias of T cell responses towards a more specialized T cell pool that results in the narrowing of the T cell repertoire.

The study of the fluctuation of the PBMC population during primary and secondary BTV infections revealed that after primary infection the number of PBMC declined significantly, whereas those numbers increased after secondary and tertiary infections. The early decline of PBMC during primary BTV infection support previous results where a high proportion of apoptosis in PBMC was observed during the peak of viremia [23]. In fact, our data show that a high number of PBMC are BTV-infected, which presumably triggers apoptosis in these cells [35]. When the virus is no longer detected in PBMC, the population expands after secondary and tertiary recall responses, supporting the hypothesis that BTV might induce PBMC apoptosis at early stages during infection. This becomes especially relevant during acute infections in which a potent T cell response is needed to mediate viral clearance. PBMC infection leading to cell death could thus dampen the immune response and promote BTV survival.

We have outlined differences between CD8^+^ and CD4^+^ T cell expansion after primary and secondary responses. CD4^+^ T cells, which help to promote B cell antibody production and are required for the generation of cytotoxic and memory CD8^+^ T cells (reviewed in [36, 37]), did not significantly increase in numbers during the time course studied here, although they displayed a trend towards an increase. This lack of significant CD4^+^ T cell expansion may be the consequence of a slowly controlled infection, or even a lack of control due to serial infections. This possible scenario in which high viral loads impair virus-specific CD4^+^ T cell responses has been reported for other viral infections [38, 39]. For BTV, the viral load at the peak of primary infection (D7pi) is very high (*Ct* values < 25), and is kept low or medium (*Ct* value > 30–40) after recall responses and until the end of the experiment. Thus, we speculate that although the viral load may not be high enough to completely impair virus-specific CD4^+^ T cell responses, our data suggest that this low/medium viral load (antigen load) is affecting the expansion of CD4^+^ T cells. The typical contraction phase of CD4^+^ T responses after acute viral infection was not detected in our experiment, in which the CD8:CD4 T cell ratio was stabilized after primary infection and never decreased. Nevertheless, it is also plausible that these data reflect a rapid contraction phase combined with ongoing CD4^+^ T cell recruitment to lymph nodes. By contrast, the kinetic of CD8^+^ T cells displays an expansion by D15pi followed by a contraction phase and expansions after secondary infections. Our results support previous work in which the rate of proliferation was similar for naïve and memory CD8^+^ T cells [3, 40]. In addition, our data show that CD8^+^ T cell responses in acute infection reached their highest responses by D15pi, which indicates that optimal CD8^+^ T cell expansion could require the presence of antigen for at least 12 days following BTV infection. For memory response upon challenge, CD8^+^ T cells initiate division and IFN-γ production with a significantly reduced duration of antigen exposure when compared to naïve T cells [41]. By D7 post-secondary infection CD8^+^ T cells already expand and produce IFN-γ. Our data thus indicate a clear activation of CD8^+^ T cells upon secondary challenge with BTV, due to the activation of memory CD8^+^ T cells that may, ideally, cross-react [21] between previously and newly encountered BTV strains.

In many viral infections, bulk T cell responses correlate weakly with the control of virus replication, whereas T cell responses to subdominant epitopes can play an important role in limiting infection [42, 43]. Moreover, antigen dose can also modify the immunodominance hierarchy of T cell epitopes [44], and thus repeated infections (and thereby increased antigen exposure) could alter the antiviral T cell response. We have characterized the evolution of the TCR repertoires specific for the VP7 protein from acute to secondary homologous BTV infection. Acute and secondary infection with BTV induces very broad repertoires in which T cell responses target a relatively high number of epitopes in VP7 (Figure 4). By contrast, after a third BTV challenge, a profound narrowing of the TCR repertoire was observed, in which 3 MHC-I and 2 MHC-II epitopes were detected. The possibility of clonotyping and/or determining the TCRβ chain usage [45] of the narrowing anti-BTV T cell repertoire could prove useful to further understand T cell responses to BTV infections in future work. In conventional vaccine design, the most immunogenic epitopes have been chosen to generate a high number of T cells directed against these dominant epitopes, and thus the T cell repertoire tends to be focused on a few epitopes [42, 46]. However, our data show that a broader T cell repertoire is initially raised to fight the incoming virus but that after recurrent infection the virus is now more likely to select and drive the activation of a more focused T cell repertoire. These findings raise the interesting possibility that a broad anti-BTV T cell repertoire may induce a faster clearance of BTV infection than a narrowly focused T cell response.

Recombinant vaccines expressing BTV subunits could therefore prove useful to trigger a broad T cell repertoire against BTV. For instance, vaccination with a recombinant adenovirus vaccine expressing VP7 partially protected sheep from a virulent challenge [12]. Adenoviral vectors can induce CD4^+^ T cell mucosal immunity and strong memory CD8^+^ T cell responses to the transgene they express [47–50]. The recombinant vaccine expressing VP7 induced a CD4^+^ T cell response and a robust CD8^+^ T cell response which was likely responsible for the control of BTV after virulent challenge. These recombinant vaccine platforms that express BTV subunits and adequately activate T cells could therefore be ideal to broaden and induce long-term memory T cell responses.

The study of the inflammatory response after sequential BTV infections revealed interesting data. IL-6, a cytokine mainly produced by activated monocytes, with pleiotropic action affecting the functions of a variety of immune cells (reviewed in [29]), is up-regulated during primary and secondary responses, and declines after the third infection. IL-6 synthesis is triggered by pathogen-recognition receptor engagement [29], and probably induced by BTV dsRNA recognition by the cell. IL-6 induces the production of the cytokine VEGF (vascular endothelial growth factor), among others, leading to angiogenesis and increased vascular permeability [51]. BTV infection is characterized by increased vascular permeability and endothelial cell dysfunction, leading to hemorrhages and edema [52]. Therefore, the high levels of IL-6 found during primary and secondary BTV infections might contribute to BTV-induced vasoactive disease. IL-6 is nonetheless also important for the development of adaptive immunity and its upregulation could help combat the infection. IL-6 promotes T follicular cell help and thus favors antibody production [53]. Moreover, it has been linked to the differentiation of activated B cells into antibody-producing plasma cells and the differentiation of CD8^+^ T cells into cytotoxic T lymphocytes [29].

Intriguingly, BTV-8 infection only induced IL-1β expression after the third viral challenge. Our results are in accordance with others, which have reported that BTV-1 primary infection triggered IL-1β expression but BTV-8 inoculation failed to induce the production of this cytokine [54]. The pro-inflammatory cytokine IL-1β is produced mostly by monocytes, macrophages and dendritic cells after pattern recognition receptor engagement and inflammasome activation [55, 56]. IL-1β promotes the recruitment of inflammatory and immune competent cells to the inflamed tissue. It is also essential for T cell-dependent antibody production [57], a pathway that BTV disrupts during infection [58]. Blockade of IL-1β activation could thus facilitate BTV survival. The increase in IL-6 detected during the first and second challenge could also contribute to impaired IL-1β activity. IL-6 triggers the production of the IL-1 receptor antagonist IL-1Ra that blocks IL-1β and IL-1α signaling [56, 59]. IL-1Ra induction by IL-6 can protect mice from autoimmune disease [59]. Whether the high IL-6 levels detected after BTV-8 infection impairs IL-1β activity will nonetheless require further investigation.

IL-12 is also a pro-inflammatory cytokine produced by B cells, macrophages and dendritic cells in response to infection (reviewed in [31, 60]). IL-12 induces IFN-γ production by T cells and drives Th1 differentiation. CXCL10 is a chemoattractant for monocytes, T cells and NK cells towards inflamed areas (review in [32]). Our data were consistent with a positive feedback loop between IFN-γ-producing Th1 cells that induce CXCL10 production on resident cells, which in turn enables CXCL10 to attract and recruit more Th1 cells.

In conclusion, BTV-8 infection induces an inflammatory response in the host characterized by increased IL-6, IL-12 and CXCL10 levels, but IL-1β levels only increased after the third infection. Our data indicate that BTV-8 infection induces a memory T cell response to subsequent homologous BTV challenges. BTV-8 re-infection also produced a narrowing of the T cell repertoire to VP7. This narrowing T cell repertoire that responds better and faster to homologous BTV challenge is, however, not sufficient to eradicate viral load. The induction and maintenance of a diverse anti-BTV T cell repertoire may therefore be more beneficial for BTV control. These findings could have implications for the design of serotype-cross-reactive BTV vaccines.
